# Supplementary figures and images for: Solubilization of skin collagen improves the accuracy and reliability of stable isotope measurements
Source: PeerJ. 2025 Oct 8;13:e20152. doi: 10.7717/peerj.20152 (PMC12514997; doi:10.7717/peerj.20152)

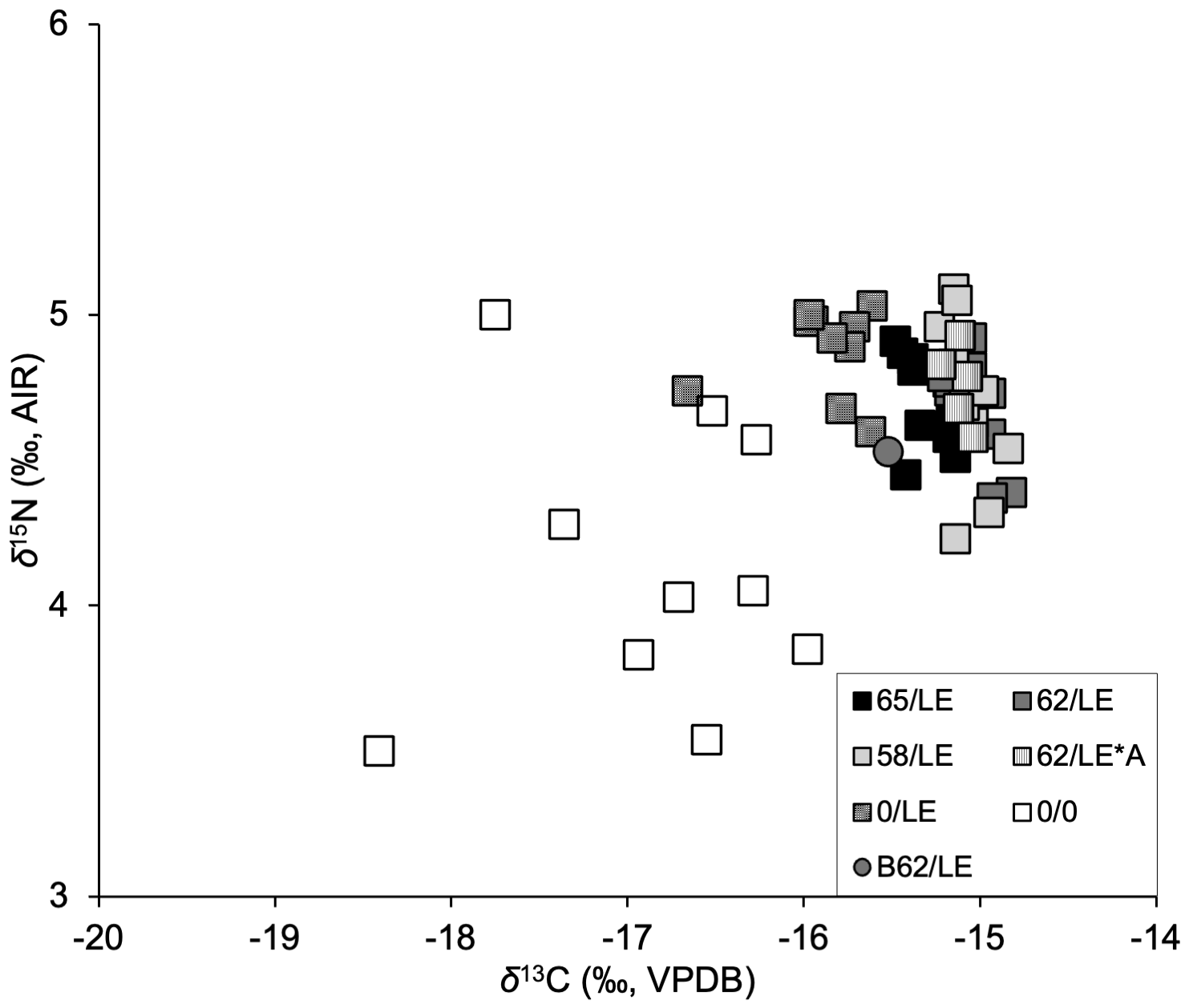

Supplement: Supplemental Information 1 [file peerj-13-20152-s001.png]

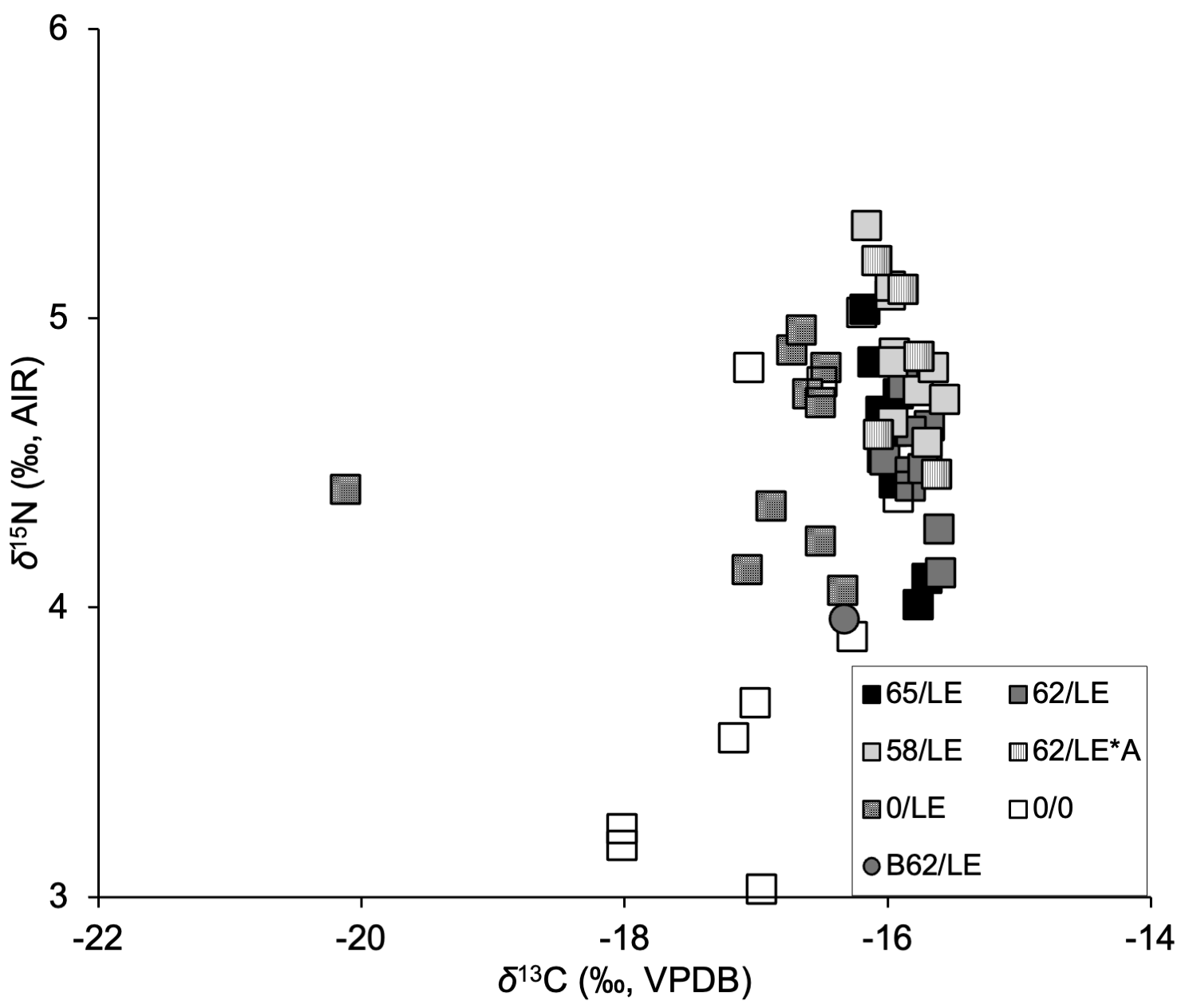

Supplement: Supplemental Information 2 [file peerj-13-20152-s002.png]

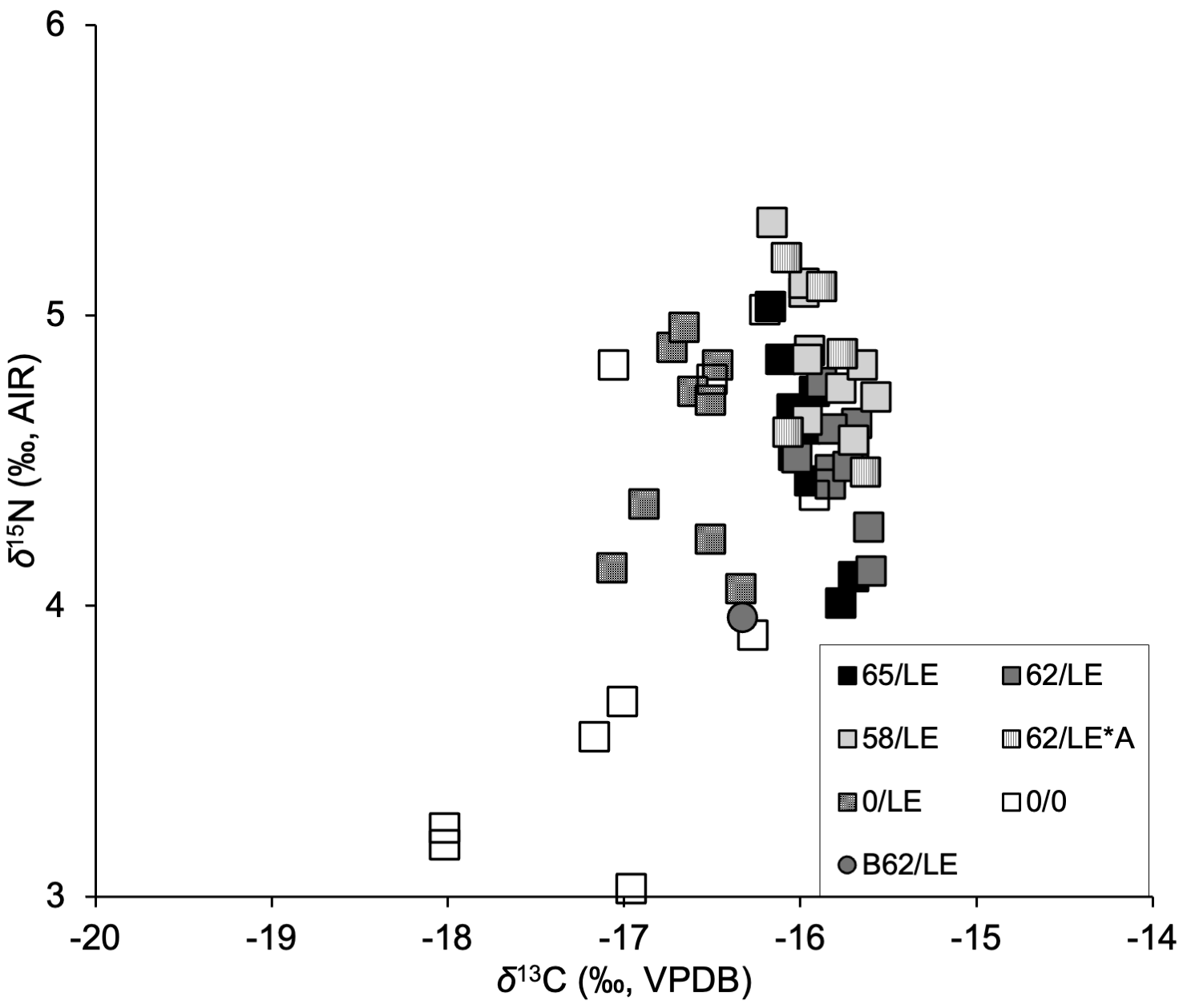

Supplement: Supplemental Information 3 [file peerj-13-20152-s003.png]
